# The Use of Cocaine in Dentistry

**Published:** 1885-09

**Authors:** J. H. Martindale

**Affiliations:** Minneapolis


### The Use of Cocaine in Dentistry.

J. H. MARTINDALE, M.D., D.D.S., MINNEAPOLIS.

*Gentlemen of the Minnesota State Dental Society:*

In attempting for a few minutes to enlist your attention on the subject of the uses of cocaine in dental surgery, I have in mind the very voluminous reports of its use that have greeted your eyes during the last few months from the pages of the dental, medical, and even the secular news.

And I feel like paraprasing the words of the celebrated opthalmologist, Dr. St. John B. Roosa, of New York, whom it was my privilege to hear expatiate on the virtues of the drug before a medical society this winter. Said Dr. Roosa, "The loneliest man on earth is the opthalmologist who has not written a paper on cocaine."

The next most lonely man in the universe we will admit is the enthusiastic dental surgeon who has not succumbed to the influence of the last tidal wave, and lent the record of his pen or raised his voice in argument or praise on the uses of cocaine in his profession.

With due allowance for the clamor that always invests with undue and exaggerated excellences any new agent, and for the over-praise of zealots and enthusiasts, it shall be our task to endeavor to present the evidences of the uses of this wonderful drug in its application to the needs of our specialty by citing cases in our own practice and in that of others, showing candidly, where our efforts have failed, and endeavoring to incite in all a determination to investigate for themselves and ascertain the best means for the application of this agent to secure our end. What is this end we seek? Briefly, it is a means whereby we may safely and readily secure to our suffering patients partial or complete immunity from the excruciating pains that attend our operations, a boon you will admit of great magnitude; for in none of the minor surgery is so much pain inflicted, and in none of the operations of surgery is it so inexpedient and difficult to effect general anæsthesia as in the case of operations in the mouth. To cocaine, as applied in the hands of the general surgeon, and to other specialties beside our own, we will give but cursory notice; suffice it to say, that within the few months that the local anæsthetic action of the drug has been known, it has become an indispensable in the armamentarium of the opthalmologist, enabling him to perform the operation strabismus, cataract, iridectomy, the removal of foreign objects, etc., without general anæsthesia, and without pain, moreover in the opthalmic operations, it is often acting at once, by virtue of its three distinct virtues, viz: anæsthetic, mydriatic, and hæmostatic, each one giving its quota to the needs of the surgeon. As a mydriatic, it acts more speedily, but its effects are more transient than in the case of atropia; as a hæmostatic its action is very marked, it constringes the capillaries quickly and strongly making operations quite bloodless, which ordinarily are unpleasantly, even dangerously bloody. Dr. W. C. Jarvis, the New York laryngologist, assured me that the removal of enlargements and certain tumors of the turbinated bones, an operation usually attended by copious and often dangerous hemorrhages, was now daily performed by him by bathing the parts with a solution of the hydrochlorate of cocaine, the operation then being effected with but little if any loss of blood. The dental surgeon, gynæcologist, and the specialist in diseases of the genito urinary organs, ascribe also to this agent new and marvelous power. In speaking in this paper of cocaine we shall, when no qualifying word is used, refer to the muriate (or hydrochlorate of cocaine.) Cocaine itself is an alkaloid derived from the leaves of a Peruvian tree of the species Coca Erythroxylon, in very much such a manner as quinine is obtained from the bark of the cinchona tree. Cocaine is a powder consisting of inodorous, colorless, and transparent prisms, of alkaline reaction and bitter taste and very sparingly soluble in water (one part in seven hundred and four of cold water.)

In consequence of its insolubility in water the alkaloid is very little used, The salts of cocaine, the muriate, oleate, citrate, and more lately the hydrobromate, (which are freely soluble in water) are for the most part the preparations in common use, in this paper we will confine ourselves to trials with the aqueous solution of the muriate and citrate, the oleate and also the above used in conjunction with other accessory drugs. Early in December of 1884, I secured in New York City some of Foucar's four per cent. solution of the muriate of cocaine and commenced a series of experiments with a view of determining the efficacy of the drug in surgical operations in the oral cavity and for the extraction of teeth. I had previous to this convinced myself that the application of various solutions directly to the cavity in carious teeth as an obtundant to allay their sensitiveness, was so ineffective in a majority of cases and so slightly effective in any, that I felt no incentive to longer continue in that track. Having learned from surgeons who were using the drug extensively hypodermically, that its anæsthetic impression (when applied in the course of a nerve) was transmitted to the nerve terminals, the idea seized me of introducing by a hypodermic injection cocaine upon the inferior dental nerve at the point where it entered the posterior dental canal; the result of my experiment was announced in the February number of the *Dental Cosmos* of this year and was briefly as follows: The injection was attempted over the nerve where it enters the dental foramen, on the internal surface of the ramus of the inferior maxilla, on the left side. The introduction of the solution (a four per cent. preparation of Foucar's) was made by Dr. Frank Hartly, of Roosevelt Hospital, of this city, and was accomplished by his first feeling for the prominent spine that guards the opening of the foramen, and to which is attached the internal lateral ligament of the lower jaw, and inserting the needle point at the place thus indicated. Twelve minims was first injected, a drop of the solution being impelled ahead of the needle as it was pushed through the mucus membrane, to render its introduction less painful.

The result that ensued, although not exactly of the character sought, was yet marked and of interest, cerebral effects were soon manifest, in considerable excitation of mind, and a pleasurable degree of warmth was experienced. Upon lancing the gums on the left side, in three or four minutes it was noticed that sensibility of the membrane was almost entirely absent on the lingual aspect, and more slightly so on the buccal; the lower lip was also benumbed, simultaneously with these phenomena, the right side of my tongue became stiff and numb; swallowing was accomplished with effort and the sense of taste on the left side almost completely lost, proving that our injection had taken effect on the lingual more than on the dental branch.

At the end of ten minutes twelve minims more was introduced at the same place, with the result only of accentuating the aforementioned action of the drug; at this junction Dr. J. S. Converse, of Thirty-seventh street, attempted the removal of an offending wisdom tooth on the left side below, but as soon as he commenced traction I called to him to desist, as it pained.

Our experiment was not a success, therefore, as regards the anæsthetizing of the inferior dental nerve in this instance, but quite so as respects the lingual branch.

Later, by means of a curved hypodermic needle, I was able to pull the needle into the posterior dental canal and hoped thereby to concentrate the influence of my injection upon the inferior dental nerve alone, avoiding the lingual and mylohyoid nerves, which are given off just before the inferior dental nerve, for I found that the impress of the drug upon the lingual and mylohyoid nerves gave rise to such unpleasant consequences as to strongly militate against the advantage sought by my experiment. Later, by efforts made by Dr. Hartley in his hospital practice he demonstrated the possibility of performing various operations on the jaws, such as the removal of teeth, tumors, etc., but as a practice in effecting anæsthesia of the parts for the purpose of dental surgery, I have given it up. But by the use of an injection into the gums of a few minims of the muriate of cocaine, I find that I am able to effect the extraction of the single-rooted teeth, and sometimes with a little or no pain. The introduction of the needle I am accustomed to render easy by first applying to the gums some of the oleate of cocaine. I trust to have an opportunity later of demonstrating before you clinically my use of cocaine for this purpose. In the case of alveolar abscesses that persist after every care has been used to carefully fill the roots of dead teeth, my practice has been to bore into the alveolus with the bur, with the dental engine, cutting away the diseased and pus-infiltrated bone, and endeavoring to tear away the abscess sack. This operation I am able to effect very easily, in case there is a fistulous opening in the alveolus, by first introducing with a hypodermic syringe as far up into the fistula as possible, and into the diseased region, a few drops of the muriate of cocaine. The scantiness of hemorrhage in this operation is very noticeable, and makes it very much easier for us to do our work, undisturbed by the obscuring of the wound by the continual oozing of blood, which is usually quite annoying. In the removal of deep-seated tartar, where the pain is considerable in the fitting of crowns by bands, and even in the application of ligatures, where we would obviate the pain caused thereby, an application of a little of the oleate of cocaine upon the gums with a camel's-hair pencil will enable us to save our patients much pain.

In a case that lately came under my charge, I had endeavored, by the use of arsenious acid, to devitalize the pulp in an inferior first molar, but upon endeavoring to extirpate the pulp, I found that the posterior root contained a vital pulp. The facts that our time was valuable (the patient being obliged to leave town), combined with a distaste I have for persisting in the use of arsenious acid, or even using it at all, induced me to introduce a drop of the oleate of cocaine into the cavity, and wait for five minutes. Upon endeavoring after that time to remove the live pulp, I was gratified to be able to do so without causing my patient the slightest pain.

In the many repeated efforts that I have made to obtund sensitive dentine as a means to enable me to more easily prepare a cavity, I am not able to make a glowing report. I made many efforts with the acqueous solutions of the muriate of varying strength, the oleate, and also the citrate. Dr. J. S. Marshall, of Chicago, reports many cases of complete anæsthesia of sensitive dentine effected by the use of little pellets of the citrate, placed in the cavity and dissolved there with a drop of water. I have tried this plan several times in cases favorable for its use, and carefully observing the course of procedure suggested by Dr. Marshall, but in no instance have I been able to secure results commensurate with the outlay of time required for the drug to act, and the fact that from the use of many other easily obtainable agents I have been able to obtund sensitive dentine more effectually and speedily.

Having now, gentlemen, assailed your ears, or, perhaps, offended your sense by a paper that is more voluminous than interesting, I shall close, submitting to my brethren in the State Society, and our distinguished visitors, the subject, having faith that in the discussion that may ensue, features of interest and value may be revealed.

